# Technology of Two-dimensional Bioimpedance Analysis of the Human Body Composition

**DOI:** 10.2478/joeb-2021-0004

**Published:** 2021-11-20

**Authors:** S. P. Shchelykalina, D. V. Nikolaev, V. A. Kolesnikov, K. A. Korostylev, O. A. Starunova

**Affiliations:** 1 SRC “Medas”, Moscow, Russia; 2 Department of Medical Cybernetics and Computer Science, Pirogov Russian National Research Medical University (RNRMU), Moscow, Russia; 3 Department of Analysis of Population Health Statistics, Central Public Health Research Institute of the Ministry of Health of Russia, Moscow, Russia

**Keywords:** bioimpedance analysis of human body composition, bioimpedance vector analysis, centile estimates, graphical representations of data, BIVA, 2DBIA

## Abstract

The BIA primary result sheets as a rule contain one-dimensional graphical scales with a selected area of normal values. In 1994, Piccoli *et al*. proposed BIVA, an alternative form of BIA data presentation, where two bioimpedance parameters are considered simultaneously as tolerance ellipses: resistance and reactance normalized to height.

The purpose of this study is to develop an approach to data analysis in body composition bioimpedance research in two-dimensional representations.

The data of 1.124.668 patients aged 5 to 85 years who underwent a bioimpedance study in Russian Health Centers from 2009 to 2015 were used. Statistical programming in the R Studio environment was carried out to estimate two-dimensional distribution densities of pairs of body composition parameters for each year of life.

The non-Gaussian distribution is found in most parameters of bioimpedance analysis of body composition for most ages (Lilliefors test, p-value << 0.0001). The slices of the actual two-dimensional distribution pairs of body composition parameters had an irregular shape. The authors of the article propose using the actually observed distribution for populations where numerous bioimpedance studies have already been carried out. Such technology can be called two-dimensional bioimpedance analysis of human body composition (2DBIA). The 2DBIA approach is clearer for practitioners and their patients due to the use of body composition parameters in addition to electrical impedance parameters.

## Introduction

The development and practical application of the technology of bioimpedance analysis of human body composition (BIA) began four decades ago. The forms of BIA data presentation have improved and developed: the result sheets have been repeatedly supplemented with new parameters, the ranges of normal values have been refined, the age range of the subjects has been expanded, and centile evaluations have been used.

**Fig. 1:**
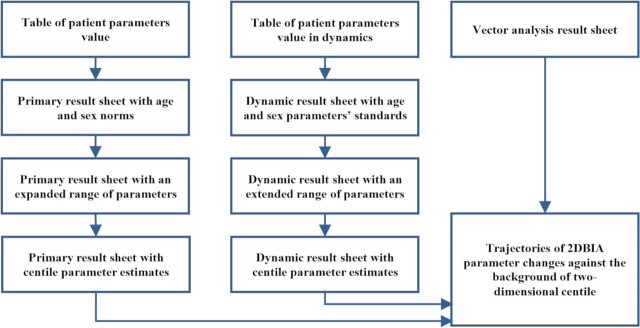
Main stages of the BIA result sheets presentation evolution.

Figure 1 shows the principal stages in the development of visual representations of BIA result sheets.

The tabular representation of bioimpedance estimates of body composition parameters in both static (for one-time examination) and dynamic (for numerous examinations of one person) BIA result sheets have, naturally, evolved into a graphical one. The current representation of the primary BIA result sheets demonstrates the geometric position of the current value markers of the BIA parameters against the background of graphic scales with gradations of “normal”, “below normal”, and “above normal”. The boundaries of the normal value area for each parameter were repeatedly revised, as a result of obtaining more and more representative samples of experimentally obtained data and localized for ethnic populations. A similar trend can be observed in dynamic result sheets: the appearance of graphs and diagrams presented against the background of norm zones and centile curves. Further development and sophistication of BIA result sheets has continuously increased the saturation of information and the speed of data reception by the physician [1].

The number of parameters calculated for body composition analysis in modern analyzers has slightly increased. In addition to the traditional estimates of body fat mass (FM), fat free mass (FFM), total, extra and intracellular fluid (TBW, ECW and ICW, respectively), estimates of active cell mass (ACM), skeletal muscle mass (SMM), and mineral mass (MM) have been added. Metabolic correlates have also been added to the list of calculated parameters: basal metabolic rate (BMR) and phase angle (PA) [2].

According to early 2000–2010 publications, samples of hundreds of thousands of people have already been received in some countries [3,4,5,6]. On the basis of this data, the boundaries of normal value areas for each parameter of body composition are created and specified, localized for ethnic populations. In the Russian Federation, samples of 820,000 and more than 1,600,000 people were collected in the Federal State Health Centers between 2009 and 2014. On their basis, the centile tables of parameters of the body composition of Russian residents have been drawn up [7].

The result sheets of a number of body composition analyzers consider pseudo-multidimensional visual representations. For example, the Tanita analyzer result sheets (https://www.tanita.com, [8]) show five-ray muscle and fat diagrams with value of the trunk, arms and legs. Such representations can include values of the upper and lower limits of the norm for a particular individual and are convenient for multidimensional representations of one individual or for estimating the balance of several indicators.

When it comes to the representation of a pair of parameters of several individuals, or trajectories developing in time of the processes of one or more individuals, flat two-dimensional graphs become a convenient representation. Two types of two-dimensional graphs can be distinguished: a graph of change in the BIA parameter value from time to time and a graph of change in the BIA parameter value from another BIA parameter, i.e. the graph where both abscissa and ordinate are represented by BIA parameters. The first bioimpedance two-dimensional representation of the parameter-by-parameter type was proposed in 1994 by Piccoli *et al*. [9]; bioimpedance vector analysis (BIVA). In BIVA, only resistance and reactance, normalized to height (H) are considered. The data from each measurement is displayed by points on the plane in the coordinates of R/H—horizontally (where: R is the resistance, H is the height of the subject) and Xc/H—vertically (where Xc is the reactance value). The results are compared with population data presented in the form of systems of embedded, concentrically arranged tolerance ellipses. It is believed that in this coordinate system, ellipses 50, 75, and 95 of centiles remain almost unchanged in the age range from 18 to 55 and can be used to analyze adult population data from.

Flat two-dimensional representations of the parameter-by-parameter type can be considered as projections of the multidimensional space of all bioimpedance parameters on the plane of two of these parameters. Other pairs of bioimpedance parameters can be used to construct two-dimensional representations, including body composition parameters widely used in clinical practice and easily, unlike BIVA, interpreted by a doctor. Such technology may be named two-dimensional bioimpedance analysis of human body composition (2DBIA).

The purpose of this work is to develop an approach to the analysis of bioimpedance studies of body composition in two-dimensional representations.

## Materials and methods

**Fig. 2:**
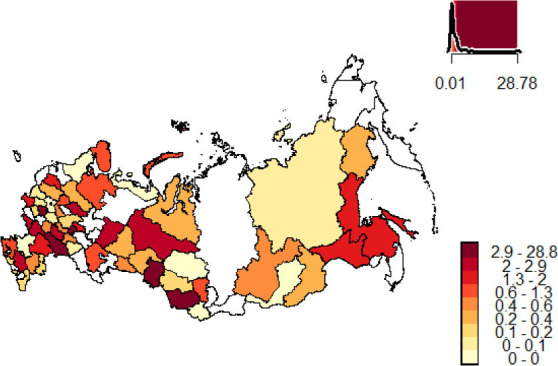
Distribution of data by subjects of the Russian Federation as a percentage of the total volume of analyzed data.

This study uses the data of bioimpedance studies of body composition accumulated in the Federal Information Resource of Health Centers (FIR HC), which was accessed within the project of RSF Grant No. 14-15-01085. The criteria for data inclusion in the studies were the age of the patient from 5 to 85 years old inclusive, the BIA conducted as a part of the patient's visit to the Health Centre between 2009 and 2015, compliance of the patient's data with the correctness criteria specially developed for the analysis of the Health Centres’ data and stated in the works of Rudnev S. *et al*. [10, 11]. All patients underwent a one-time bioimpedance study of body composition using the ABC-01 Medas device (SRC “Medas”, Russia, Moscow) and measurement of body weight and height using an automatic scales and height meter as part of the “Health-Expess” hardware and software complex (Medical Computer Systems Ltd, Russia, Moscow, Zelenograd). The probing current frequency of the bioimpedance analyzer is 5 and 50 kHz. Data from 311 Health Centers located in 61 federal subjects was used (fig. 2). A total of 1,124,668 patients were included in this study. Descriptive statistics of the data are presented in the form of medians and inter-quantile range in Table 1. The analysis included studying the distribution of the bioimpedance analysis data of the Russian population in different age categories using normal Q-Q plots, Lilliefors (Kolmogorov-Smirnov) test, energy tests of multivariate normality.

**Table 1:** Median and quartile values of the main bioimpedance parameters used

	Total		Children (5–17 years old)		Adult (18–85 years old)	

Sex, N	Men 367,593	Women 757,075	Boys 185,927	Girls 174,815	Men 181,666	Women 582,260
Age, years	17.8 [12.2; 44.9]	43.8 [19.4; 58.2]	12.3 [9.2; 15.3]	12.4 [9.2; 15.5]	45.3 [29.3; 58.4]	51.6 [36.9; 61.2]
Height, cm	170 [153; 177]	160 [155; 165]	153 [136; 171]	154 [135; 163]	175 [170; 179]	161 [157; 165]
Weight, kg	67 [46; 82]	66 [54; 79]	47 [32; 62]	45 [31; 55.2]	80 [71; 91.6]	71 [61; 83]
BMI, kg/m^2^	22.8 [18.8; 27.2]	25.6 [20.9; 30.7]	19.1 [16.6; 22.1]	18.9 [16.4; 21.7]	26.6 [23.5; 29.9]	27.6 [23.4; 32]
R50, Ohm	539 [473; 635]	586 [520; 663]	623 [545; 702]	696 [632; 764]	484 [440; 533]	560 [503; 622]
Xc50, Ohm	64 [57; 72]	66 [57; 76]	67 [61; 75]	75 [67; 83]	61 [53; 68]	63 [55; 72]
Phi50, deg.	6.6 [5.9; 7.4]	6.3 [5.8; 6.9]	6.2 [5.7; 6.8]	6.1 [5.6; 6.7]	7.1 [6.4; 7.8]	6.4 [5.9; 7]
R5, Ohm	677 [591; 764]	730 [638; 815]	695 [614; 775]	772 [700; 844]	543 [479; 599]	623 [547; 694]
FFM, kg	53.9 [35.7; 62.3]	44.2 [39.5; 48.9]	36.0 [25.7; 49.5]	33.6 [24.2; 40]	61.4 [56.3; 67.1]	45.8 [42.2; 50.1]
FM, kg	13.2 [7.4; 21.1]	21.7 [13.9; 30.8]	8.6 [5.2; 13.5]	10.9 [6.2; 16]	19.1 [13.0; 26.0]	25.2 [17.9; 33.3]
FM, %	21.5 [15.9; 27.6]	33.1 [26.0; 39.3]	19.2 [14.6; 25.3]	25.0 [19.4; 30.3]	23.9 [18.1; 29.0]	35.5 [29.2; 40.8]
SMM, %	51.6 [48.3; 55.7]	43.7 [39.9; 47.1]	54.9 [48.5; 58.6]	45.2 [35.5; 49.2]	50.3 [48.3; 52.7]	43.5 [40.4; 46.4]
TBW, l	39.4 [26.1; 45.6]	32.4 [28.9; 35.8]	26.4 [18.9; 36.3]	24.6 [17.9; 29.3]	44.9 [41.2; 49.1]	33.6 [30.9; 36.7]
ECW, l	12.6 [9.3; 16.2]	12.2 [9.7; 14.1]	11.6 [9.0; 15.2]	11.0 [8.6; 12.8]	17.9 [16.2; 20.0]	14.3 [12.9; 16.1]
ICW, l	15.0 [9.7; 22.3]	15.4 [10.2; 18.6]	13.5 [9.3; 20]	12.7 [8.6; 16]	27.5 [25.0; 30.1]	19.1 [17.6; 20.8]
ACM, kg	30.7 [19.2; 36.7]	24.5 [21.5; 27.5]	19.4 [13.7; 27.9]	18.2 [12.9; 22.4]	36.1 [32.4; 40]	25.5 [23.1; 28.3]
iFM, kg/m^2^	4.8 [3.0; 7.3]	8.4 [5.4; 11.9]	3.5 [2.5; 5.4]	4.7 [3.2; 6.4]	6.3 [4.3; 8.5]	9.7 [6.9; 12.9]
iFFM, kg/m^2^	18.0 [15.2; 20.4]	17.1 [15.2; 18.9]	15.3 [13.7; 17.1]	14.1 [12.9; 15.6]	20.2 [18.7; 21.9]	17.8 [16.3; 19.4]
BMR, kcal/m^2^	1586 [1224; 1775]	1390 [1295; 1485]	1230 [1047; 1498]	1191 [1023; 1323]	1755 [1639; 1881]	1421 [1347; 1510]

BMI – body mass index, R50 – 50 kHz resistance, Xc50 – 50 kHz reactance, Phi50 – 50 kHz phase angle, R5 – 5 kHz resistance, FFM – fat free mass, FM – fat mass, percent from weight, SMM - skeletal muscle mass, percent from fat-free mass, TBW – total body water, ECW – extracellular water, ICW – intracellular water, ACM – active cell mass, iFM – fat mass index, iFFM – fat-free mass index, BMR – basal metabolic rate.

The null hypothesis of the normal distribution was rejected at p <0.05. Comparison of groups of rugby players and patients with diabetes and sarcopenia with the population was carried out using the Multivariate nonparametric rank test (C Sample Test of Location) [12, 13]. The analysis was carried out with the help of statistical programming in the R Studio environment using the ‘conics’ packages by Bernard Desgraupes (University of Paris Ouest—Nanterre, Lab Modal’X (EA 3454)), ‘ks’ by Tarn Duong, Matt Wand, Jose Chacon, Artur Gramacki, ‘nortest’ by Juergen Gross, Uwe Ligges, ‘energy’ by Maria Rizzo, Gabor Szekely, ‘MNM’ by Klaus Nordhausen, Jyrki Mottonen, Hannu Oja.

For practical application of two-dimensional centile representations of body composition parameters, the Animation software tool (SRC “Medas”, 2016) was developed. It used the native data to construct slices of the actual two-dimensional distribution of any selected pairs of parameters of bioimpedance evaluation of body composition and made it possible to visualize the data of a specific patient or patient group against this background. As illustrative examples, this article uses depersonalized patient data obtained with informed consent for medical purposes from the Federal State Budgetary Educational Institution of Higher Education “Yaroslavl State Medical University” of the Ministry of Health of the Russian Federation, (Yaroslavl, Russia) and the State Budgetary Institution of Healthcare of the Yaroslavl region “Regional perinatal center”, (Yaroslavl, Russia), the Federal State Budgetary Institution “Federal Bureau of Medical and Social Expertise” of the Ministry of Labor and Social Protection of the Russian Federation (Moscow, Russia), the Regional budgetary healthcare institution “Ivanovo Regional Clinical Hospital” (Ivanovo, Russia), the Federal State Budgetary Scientific Institution “Federal Research and Clinical Centre for Resuscitation and Rehabilitology” (Moscow region, Russia) and the medical center of the Russian national rugby-7 team.

### Ethical approval

The research conducted is not related to either human or animal use.

## Results

**Fig. 3:**
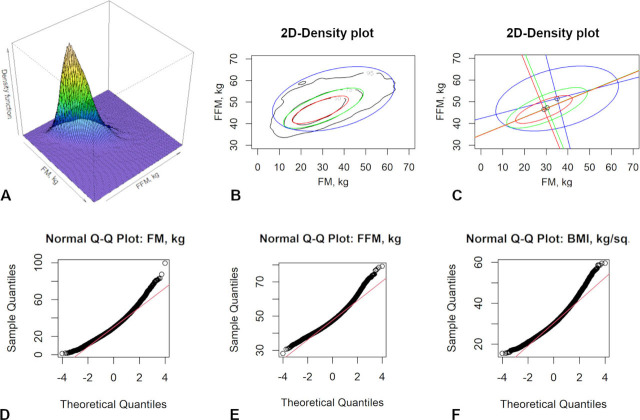
Visual representations of the two-dimensional density distribution of Fat mass and Fat free mass (A–C) and normal quantile-quantile plots (D–F) in 57-year-old women (data for 15,434): A. a three-dimensional graph of the density distribution, B. flat sections containing inside 50%, 75% and 95% of data (black lines are sections obtained directly from the data, colored lines are calculated ellipses), C. axes and centers of tolerance ellipses, D. a normal quantile-quantile plot of Fat mass, E. a normal quantile-quantile plot of Fat free mass, F. a normal quantile-quantile plot of Body mass index.

The type of distribution was assessed in sex groups for each year of life. The size of the groups ranged from 78 people (85-year old men) to 19,636 people (16-year old men). The non-Gaussian distribution is found in most parameters of bioimpedance analysis of body composition for most ages (Lilliefors test, p-values << 0,0001). Only 2% of univariate distribution type studies found a Gaussian (p-values > 0.05) for small groups of oldest-old Health Center visitors. Figure 3 shows the distribution of FM and FFM for 15,434 57-year old women. It can be clearly seen (fig. 3A) that the three-dimensional graph of the distribution density is not a regular cone, i.e. is not Gaussian and cannot be correctly represented as concentric ellipses with a single center and direction of the axes. Figure 3B shows the cross-sections of the probability density, containing 50%, 75% and 95% of the data, respectively (black lines), the colored lines represent the correctly calculated ellipses for the same centiles. It can be seen that real sections have an uneven contour, and elliptical curves do not really have a common center and axes (fig. 3C).

The same trend is present for different parameters for all ages of both male and female parts of the population. In this case the non-Gaussian multivariate distributions are explained by the high prevalence of obesity: more than 52% of the group have BMI > 30 kg/m^2^, median and quartiles of BMI are 30.8 kg/m^2^ [26.7 kg/m^2^; 34.2 kg/m^2^]. Figures 3D–3F show normal quantile-quantile plots FM, FFM and BMI and it shows that these parameters are not normally distributed (p-values << 0.0001). Energy tests of multivariate normality for different paraments pair, ages and sex found significant deviations from normality (p-value << 0.0001).

The most notable deviations from the elliptic form of dispersion in many ages were found for the 95% tolerance curve. This phenomenon is due to the large variability of rarely occurring combinations of initial parameters in the raw data and, as a result, in general, the form of data dispersion is far from elliptical. Thus, the usage of Gaussian multivariate distributions with two-dimensional elliptic representation to describe the entire population does not seem reliable enough. The volume of bioimpedance research data collected at Russian Health Centers allows us to construct a planar representation of two-dimensional distributions without using a pre-selected curve shape.

Using the specially developed Animation software (SRC “Medas”, 2016), on the basis of native bioimpedance data of more than 1.1 million people, two-dimensional centile curves for several pairs of body composition parameters were constructed. The software allows selecting any pair of parameters and setting any age range from 5 to 85 years. To assess body composition at the individual level in a single study as well as to learn about the population age dynamics of the parameters, a fairly narrow age range can be chosen, for example, within one year. This step clearly shows age-related changes in the dynamics of body composition parameters, and the typicity of individual patient indicators are evaluated accurately. Any combination of BIA parameters can be selected when drawing two-dimensional centile curves. Colored numbers in the right upper corner show population value inside the corresponding closed curves. The upper left corner displays population age and population value used for constructing each centile curve. Also, the overall age structure of data used is visible.

**Fig. 4:**
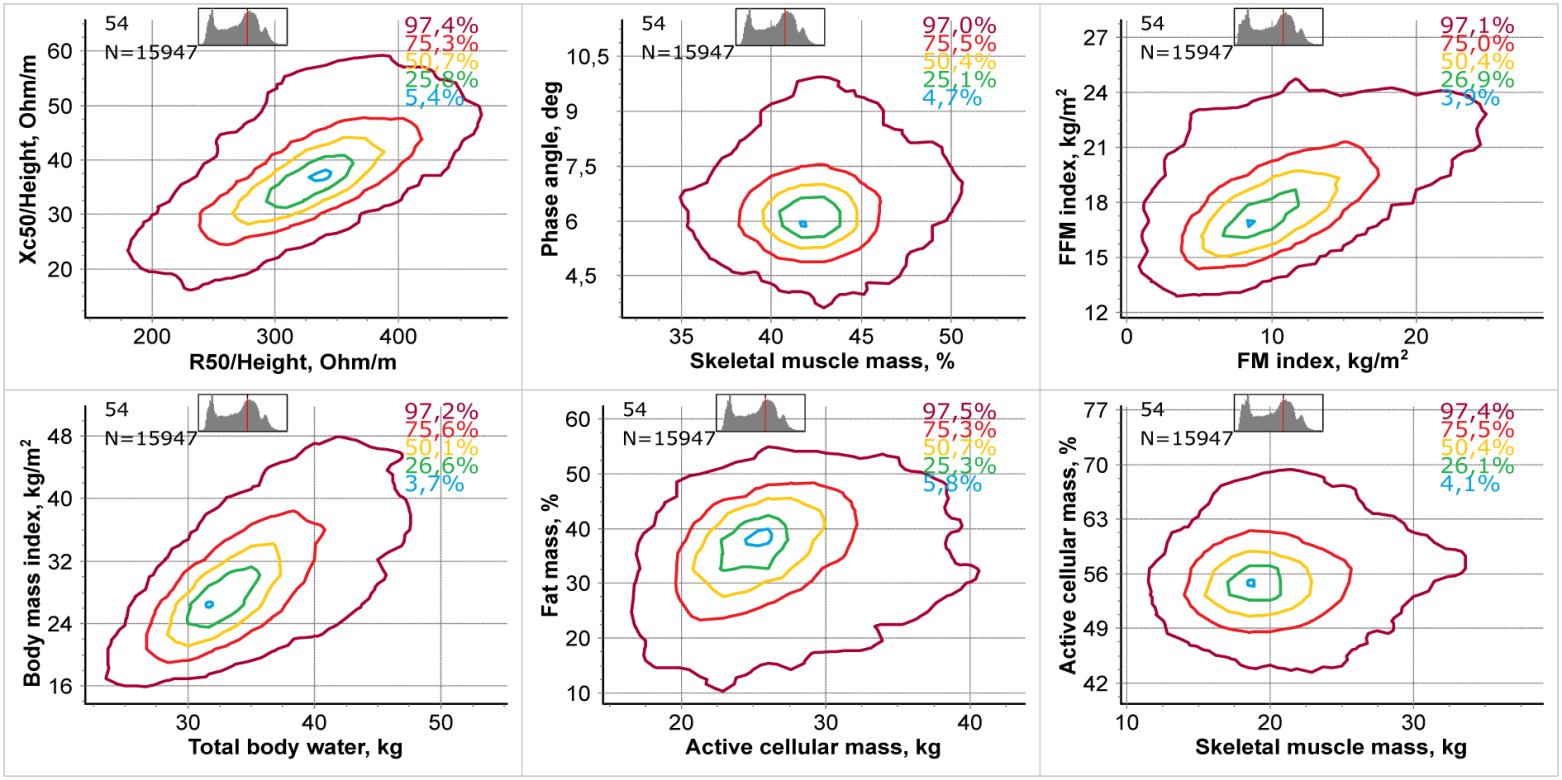
An example of visualization of a bioimpedance study on 12 parameters, with centile lines arranged in two-dimensional graphs constructed from data of 15,947 women aged 54 full years.

Figure 4 offers an example of two-dimensional centile graphs for six pairs of body composition parameters calculated from 15,947 of a sub-population of 54-year-old women. They include the following graphs: R/H and Xc/H (classic BIVA), the proportion of skeletal muscle mass in fat free mass (SMM%) and PA, fat mass index (iFM) and fat-free mass index (iFFM), TBW and BMI, ACM and the proportion of fat mass (FM%), SMM and the proportion of active cell mass (ACM%). These pairs of parameters were selected as they are the most used in cardiology, gerontology, dietetics, nutritional medicine, sports medicine, etc. Animation software allows the user to choose any combination of parameters that interests them, guided by their own professional knowledge.

It is clear that the centile graphs of all parameter pairs are not concentric ellipses and all two-dimensional distributions are non-Gaussian (Energy tests of multivariate normality, p-values << 0.0001). The observed shape of the centile curves is specific for each pair of parameters.

**Fig. 5:**
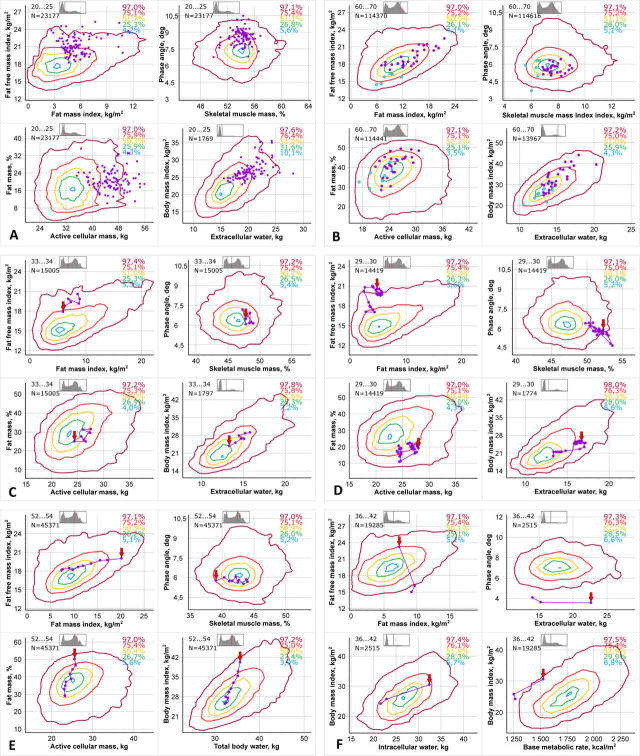
Examples of “2DBIA” visualization of bioimpedance examination by 8 parameters, with centile lines, arranged in two-dimensional graphs. Every point corresponds individual examination data. Dynamic observation of one patient is presented by line connected points. The arrow marks to the first examination point. A. Data on a group of professional rugby players, 97 men 20–25 years old. B. Data on a group of diabetic patients 60–70 years old. Pink points belong to 59 non-sarcopenic patients, blue points belong to 7 sarcopenic patients. C. Data on a healthy pregnant woman 33 years old from 11^th^ to 37^th^ weeks of gestation. The points correspond to 11^th^ (marked with an arrow), 16^th^, 22^nd^, 26^th^, 30^th^, 34^th^, 37^th^ weeks of gestation. D. Data on a pregnant woman 29 years old with a preeclampsia. Examinations were made daily from 30^th^ week of gestation to the delivery at the 32^nd^ week of gestation and on 2^nd^, 3^rd^, 4^th^, 5^th^, 8^th^, 9^th^, 10^th^, 11^th^, 12^th^ and 15^th^ day postpartum. E. Data on fat mass reduction in a 52 years old woman without any recorded associated diseases. F. Data on an intensive care unit patient with edema.

The 2DBIA centiles curves (fig. 5) were constructed for the same group of women and men of the Russian population using the Animation software. The data points of the bioimpedance study of a specific patient are connected by straight lines. The arrow indicates the point of the first examination.

The points correspond to the individual examination data for different clinical cases. The points on fig. 5A show data from 97 20 to 25 year old men who are professional rugby players. Figure 6B plots diabetic patients 60 to 70 years old with sarcopenia (blue points) and without it (pink points). Figures 5C to 5F show dynamic observations of individual patients: during 23 weeks healthy pregnancy (fig. 5C), during 2 weeks pregnancy of a patient with preeclampsia and during 2 weeks after delivery in the 32^nd^ week of gestation (fig. 5D), obesity patients in the process of reducing their fat mass (fig. 5E), an intensive care unit patient with edema (fig. 5F).

## Discussion

It is convenient to have compact visual representations of valid values for a particular clinical situation both at primary diagnostics and in the course of estimating efficiency of medical actions. As for the BIA, it is possible to talk about 6–8 parameters of body composition, characterizing the processes typical for a particular disease or the task of assessing the body's reserves. The primary result sheets use on average 12 one-dimensional, usually, graphic scales characterizing the patient's body composition at the time of the study. The presentation of patient data as a point or a set of points on a population centile picture contains additional information about the typicality of such parameter values in a given population.

In a large population study between 2014 and 2016 (Hwad: V. Starodubov) it was found that the distribution of bioimpedance data in the Russian population shows significant difference from Gaussian. The authors of the article propose using the actually observed distribution for populations where numerous bioimpedance studies have already been carried out.

Parameters of body composition, already familiar to physicians through their courses of normal and pathological physiology, can be used as a basis for a two-dimensional bioimpedance analysis of human body composition. It means the 2DBIA can help improve the quality of medical care and patient adherence to therapy. Since the parameter values of body composition bioimpedance analysis and their distribution in the population undergo significant changes with age, the image of 2DBIA, built for narrow age intervals, should be considered for clinical use.

Data visualization, containing more than two dimensions, entails difficulties in graphical construction, and, more significantly, information perception. A compromise solution is proposed to place four two-dimensional images of eight basic body composition parameters on the background of two-dimensional centile curves on one screen and on one sheet of the result sheet. The technique of constructing two-dimensional curves of 2DBIA was considered in work by Nikolaev D.V. *et al*. [14]. Figures 5 show the examples of such an image consisting of four 2DBIA images. The following pairs of main bioimpedance parameters were selected: iFFM and iFM, SMM% or skeletal muscle mass index (iSMM) and PA, ACM and FM%, ECW and BMI, TBW and BMI, ICW and BMI, BMR and BMI for different examples. Contours containing 97, 75, 50, 25, and 3 percent of data from the sex-age group were chosen as centile representations. The exact value of the amount of data contained within the contour is indicated in the upper right corner of each centile picture. It can be seen that the two-dimensional centile curves have an irregular shape, far enough from the ellipse.

Apparently, the irregularity of the curves’ shape is explained by the features and the size of the population. In this case from 20% to 80% of the Russian population depending on sex and age are overweight or obese [7]. 95% tolerance curves almost always have many bends, as it describes more varied and rare combinations of parameter values.

Figures 5 demonstrate some clinical applications of the new 2DBIA presentation. It is easy to notice that professional rugby players (fig. 5A) differ significantly (p << 0.0001) from the general Russian population of the same sex and age. On all pairs of centile curves they occupy only one quarter, the upper right square in the coordinates of iFM and iFFM, have high phase angle values and average %SMM values, high ACM values and average %FM values and high BMI values and high ECW values.

Patients with diabetes (fig. 5B) differ little from the general Russian population: most of the points lie within the 75% tolerance curves on all the presented two-dimensional centile images. In this case, patients with sarcopenia form a group separate from patients without sarcopenia. Three out of four showed two-dimensional distributions significantly diverging between patients and population (p = 0.009, <<0.0001, <0.001), and all showed two-dimensional distributions significantly diverging between patients with and without sarcopenia (p = 0.0001, 0.001, 0.012, 0.002). Distributions of BMI/ECW were not significantly different between patients and population (p = 0.506). The 2DBIA can be used for both descriptive presentation and diagnostic purposes.

Figures 5C to 5F show changes in body composition parameters in accordance with physiological and pathological changes in the body. Figure 5C shows the sequence of changes in body composition parameters typical for a physiologically proceeding pregnancy, accompanied by an increase in body weight to the accumulation of fluid in the body tissues and the growth of the fetus, ovum, placenta and uterus: from the 11^th^ to 37^th^ weeks of gestation, bioimpedance analysis registers an increase in iFM against the background of a less pronounced increase in iFFM and a pronounced increase in BMI and extracellular fluid volume. In general, there is an undeniable tendency for the indicators to go beyond the internal curves of tolerance.

A two-week follow-up examination of a pregnant woman (fig. 5D) with preeclampsia did not show any clear trend in body parameters. All this time, the parameter values fluctuated near the border of the 95% tolerance curve. Postpartum, all parameters of body composition smoothly moved from the 95% tolerance curves to the 50% or 25% tolerance curves within two weeks. The patient was discharged on the 15^th^ day.

During the development of the fetus, the fat mass experienced fictitious increases in order to increase the body weight of the woman's fetus, amniotic water and increasing uterine weight: in the lower right quadrant a sharp decrease iFM on the third day after birth (fig. 5D) is visible. In coordinates of ACM/FM% patient's parameters jumped along the 75% tolerance curve after childbirth.

Figure 5E shows data from a 2-year follow-up examination of a woman in the process of fat mass reduction. The figure shows that during the fat mass reducing process, the parameter values moved closer to the central zone, which corresponds to the most typical values for the Russian population. In the prevailing number of cases, the general direction of the broken line shows the trends of changes which are typical for decreasing fat mass values in a practically healthy patient. There is a tendency to normalize body composition parameters which results in a movement from external centile contours to internal ones.

Figure 5F shows data from a 6-week follow-up examination of a patient in an intensive care unit. The general edema was clinically apparent. During their stay in the intensive care unit, the doctors were able to remove 19.5 liters of total body water and 8.5 liters of extracellular water and stabilize the patient's state. At the time of writing, the patient is in a moderate condition and has not yet been discharged.

## Conclusion

The idea of compressing BIA data in two-dimensional representations may be useful. Visual representations of the 2DBIA allow analyzing the selected combinations of body composition parameters. It is shown that such visual representations can be used for both single and dynamic studies of body composition. A simultaneous image of several pairs of parameters allows a more complete assessment of the patient's condition and its change during a dynamic observation. Thus, the possibility of a real multivariate estimation of body composition parameters is excluded. Considering the volume of bioimpedance data accumulated in different countries, localized reference images of 2DBIA can be constructed for each sex and age, which will increase the adequacy of patient assessment and minimize the probability of errors. Time will show whether this technology will be in demand.
